# Landscape metrics as functional traits in plants: perspectives from a glacier foreland

**DOI:** 10.7717/peerj.3552

**Published:** 2017-07-31

**Authors:** Tommaso Sitzia, Matteo Dainese, Bertil O. Krüsi, Duncan McCollin

**Affiliations:** 1 Department of Land, Environment, Agriculture and Forestry, Università degli Studi di Padova, Legnaro (PD), Italy; 2 Landscape & Biodiversity Research Group, The University of Northampton, Northampton, United Kingdom; 3 Department of Animal Ecology and Tropical Biology, Universität Würzburg, Würzburg, Germany; 4 School of Life Sciences and Facility Management, Zürich University of Applied Science, Wädenswil, Switzerland

**Keywords:** Ecological process, Plant composition, Life-history trait, Pioneer plant, Landscape pattern, Spatial self-organisation, Spatial pattern, Landscape heterogeneity, Patch size, Patch shape

## Abstract

Spatial patterns of vegetation arise from an interplay of functional traits, environmental characteristics and chance. The retreat of glaciers offers exposed substrates which are colonised by plants forming distinct patchy patterns. The aim of this study was to unravel whether patch-level landscape metrics of plants can be treated as functional traits. We sampled 46 plots, each 1 m × 1 m, distributed along a restricted range of terrain age and topsoil texture on the foreland of the Nardis glacier, located in the South-Eastern Alps, Italy. Nine quantitative functional traits were selected for 16 of the plant species present, and seven landscape metrics were measured to describe the spatial arrangement of the plant species’ patches on the study plots, at a resolution of 1 cm × 1 cm. We studied the relationships among plant communities, landscape metrics, terrain age and topsoil texture. RLQ-analysis was used to examine trait-spatial configuration relationships. To assess the effect of terrain age and topsoil texture variation on trait performance, we applied a partial-RLQ analysis approach. Finally, we used the fourth-corner statistic to quantify and test relationships between traits, landscape metrics and RLQ axes. Floristically-defined relevé clusters differed significantly with regard to several landscape metrics. Diversity in patch types and size increased and patch size decreased with increasing canopy height, leaf size and weight. Moreover, more compact patch shapes were correlated with an increased capacity for the conservation of nutrients in leaves. Neither plant species composition nor any of the landscape metrics were found to differ amongst the three classes of terrain age or topsoil texture. We conclude that patch-level landscape metrics of plants can be treated as species-specific functional traits. We recommend that existing databases of functional traits should incorporate these type of data.

## Introduction

Heterogeneity of vegetation pattern has long been a subject of debate in ecology (e.g., Greig-Smith, 1979; Macfadyen, 1950). One area of focus on vegetation patterns is toward spatial self-organization, regular pattern formation arising as an emergent property of local biotic interactions in combination with large-scale physical agents especially in relatively simple ecosystems such as deserts (Rietkerk & VandeKoppel, 2008; Sole & Bascompte, 2006) or glacier forelands (Körner, 2003). Such interactions between biotic and prevalent physical agents, such as directional water drainage in peatlands or tidal currents in shallow marine beds, can generate striking spatial patterns such as stripes or polygons (Jiang et al., 2012; Van de Koppel, Bouma & Herman, 2012).

Spatial patterns are frequently associated with primary succession on glacier forelands where the barren soil is gradually colonised by plants mostly characterised by clonal growth (Körner, 2003). Small-scale interactions between organisms and physical processes can break the symmetry of bare glacial deposits to initiate pattern formation in terms of a concentration or aggregation of individuals in clusters. After glacier retreat, vegetation and soil usually develop rapidly (Chapin et al., 1994; Matthews, 1999), with changes often driven more by allogenic than by autogenic mechanisms during the very early stages of succession (Matthews & Whittaker, 1987).

Successional rates are non-linear, with the fastest increases in total vegetation cover during the first 15–20 years after deglaciation (Raffl et al., 2006) and significant local variability in species composition at the youngest sites. Accordingly, terrain age might not be the principal factor in explaining present-day variation in species composition on glacier forelands (Rydgren et al., 2014). Depending on the time range considered as well as the sampling and analytical procedures employed (Rydgren et al., 2014), species’ response may depend on factors other than time since deglaciation (Matthews & Whittaker, 1987; Těšitel et al., 2014). Amongst these, it has been shown that simple morphological and physiological attributes (i.e., life-history or functional traits) combined with suitable establishment and environmental conditions, are the most important drivers of colonization success (Erschbamer, Niederfriniger Schalg & Eckart, 2008). Such attributes are often helpful for identifying successional stages (Caccianiga et al., 2006; Erschbamer & Mayer, 2011; Nascimbene et al., 2017; Schwienbacher et al., 2012). Indeed, the integration between the available trait databases, and databases that combine the abundance of species with environmental information, can help to identify the traits that respond, influence or interact with environmental factors and ecological processes (Suding et al., 2008), a major field of functional ecology (McGill et al., 2006).

Early stage succession should be mainly driven by neutral or trait-driven dispersal processes, while competition filtering should show stronger responses in later stages. In the case of herbaceous plants, competitive effects may be found over only very small distances, not exceeding a few centimetres (Schleicher, Peppler-Lisbach & Kleyer, 2011). The study of these competitive effects on glacier forelands may be helped by a high resolution landscape ecological approach, where vegetation patterns are linked to plant trait characteristics, with the aim of revealing interactions between spatial patterns and ecological processes.

Previous analyses of vegetation patterns during glacier foreland primary succession suggest a sequence of wave-like replacements of groups of species, largely in order of increasing size (Reiners, Worley & Lawrence, 1971). Matthews & Whittaker (1987) observed that an early peak in mean diameter of *Poa alpina* L. and *Oxyria digyna* (L.) Hill clumps during succession is followed by a rapid decline in development and then in size and cover values from around 50 y terrain age. In contrast, shrubs, but also clumps of certain herbaceous species, e.g., *Saxifraga oppositifolia* L., have a tendency to increase in size and number of flowering individuals with increasing terrain age (Těšitel et al., 2014).

Recently, the combination of a three-table ordination (RLQ analysis) (Dolédec et al., 1996) and the fourth-corner method, the latter an approach to test the direct correlation between a single trait and a single environmental variable (Legendre, Galzin & HarmelinVivien, 1997), has been proposed to assess trait responses to environmental variation (Dray et al., 2014). Here, we apply these techniques, along with cluster analysis and ANOVA, to elucidate how plant species and life trait composition as well as the spatial configuration of plant species patches are related on recently deglaciated substrates.

Plant growth development following glacier retreat should lead to spatial organization in the form of discs, rings, or fragmenting clusters, very slowly moving across the landscape, like in tussock graminoids, or isolated ramets forming widely spread nets of a single genet, or stoloniferous or rhizomatous forbs (Körner, 2003). Such patterns can be measured using metrics derived from landscape ecology, e.g., numbers, size, shape, type, and the spatial arrangement of plant species patches (Forman & Godron, 1981).

Our main research question here concerns how landscape metrics correlate with measures relating to plant species composition and functional traits. We restrict sampling to terrain ages of >15 years, which would not include the very early pioneer stage, and <70 years, before the establishment of woody species. Within these constraints, we hypothesise that differences in spatial configuration would combine with plant trait patterning, while terrain age and environmental variability, intentionally reduced, should have minimal effects.

Landscape filters on functional traits have received increasing attention in recent years on both animals and plants (e.g., Deckers et al., 2004; Duflot et al., 2014; Gámez-Virués et al., 2015). In general, studies often agree that landscape-scale effects could dominate community-level filtering on functional traits (Gámez-Virués et al., 2015). Landscape metrics are usually determined at large scale resolutions, for example within a certain distance from the target habitats, e.g., hedgerows (Deckers et al., 2004) or grasslands (Gámez-Virués et al., 2015) or from land-cover maps in 1 km^2^ landscape replicates (Duflot et al., 2014). Here we adapt this approach, often used over the scales of several kilometres, to a species-level approach over scales of centimetres to metres.

We aim to provide insight into the role of plant traits in spatial organization on glacial forelands at high resolutions, but also, in general, to test hypotheses regarding the linkage between plant trait distributions and species-level spatial organization. For instance, metrics quantifying landscape complexity may be related to a higher frequency of morphological traits related to competitive ability, while metrics related to patch compactness may be related to traits that indicate conservation of acquired resources. The patch-level landscape metrics of plant individuals could be treated as functional traits themselves.

## Materials and Methods

### Study area

**Figure 1:**
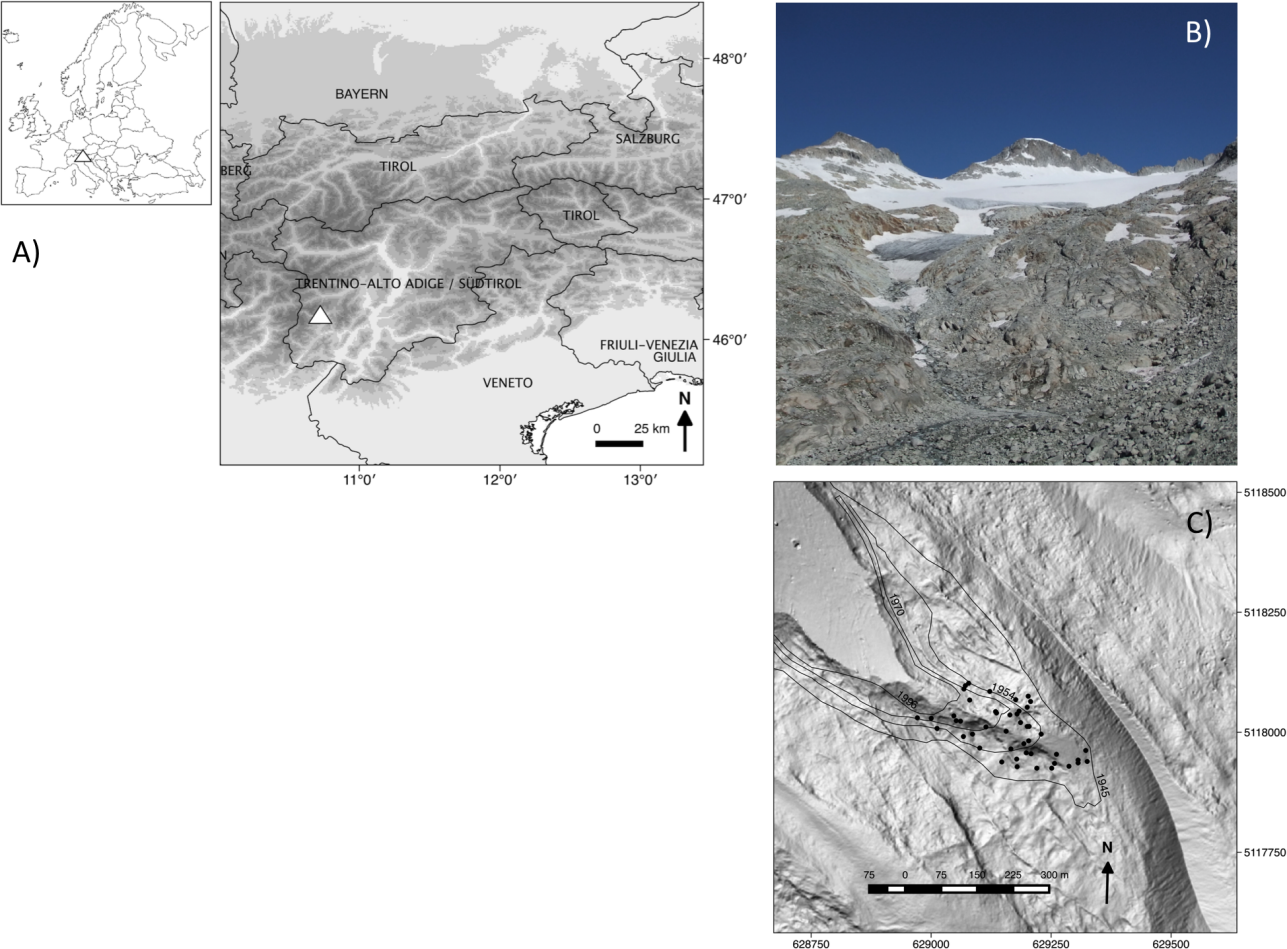
(A) Location of the study area (triangles), (B) image of the glacier foreland in 2011, and (C) a map of how the glacier has retreated from 1945 (external isochrones) to 2006 (date of the digital terrain model used as a base map), with the positions of the 46 1 m × 1 m-plots (black dots). Coordinates are reported according to the geographic coordinate reference systems: (A) WGS84 (EPSG: 4326) and (C) ETRS 89/UTM zone 32N (EPSG: 25832).

Field work was performed on the Nardis glacier foreland (46°12′14″N, 10° 40′21″E), located in the Adamello-Presanella group (Rhaetian Alps, southern sector of the Italian Central Alps) on the southern slope of Presanella Peak (3,556 m a.s.l.) (Fig. 1A). The glacier has a surface area of approximately 1.67 km^2^ (SAT, 2007) and its tongue extends down to an altitude of 2,720 m a.s.l. (Fig. 1B). The bedrock consists primarily of acidic granitoid material. The geology is characterized by the large Adamello-Presanella-Monte Re di Castello batholith (29.4–41 Ma), consisting of tonalite, an igneous, plutonic intrusive rock. Available climatic data taken from a nearby weather station (46°25′33″N, 10°41′51″E) located at the same altitude indicate a mean summer temperature of 5.7°C and a mean annual precipitation of 897 mm. The study area where the sampling took place was approximately 7 hectares in size and corresponded to the zone in front of the glacier tongue, where the glacier was still present in 1945 (Fig. 1C).

### Data collection

**Figure 2:**
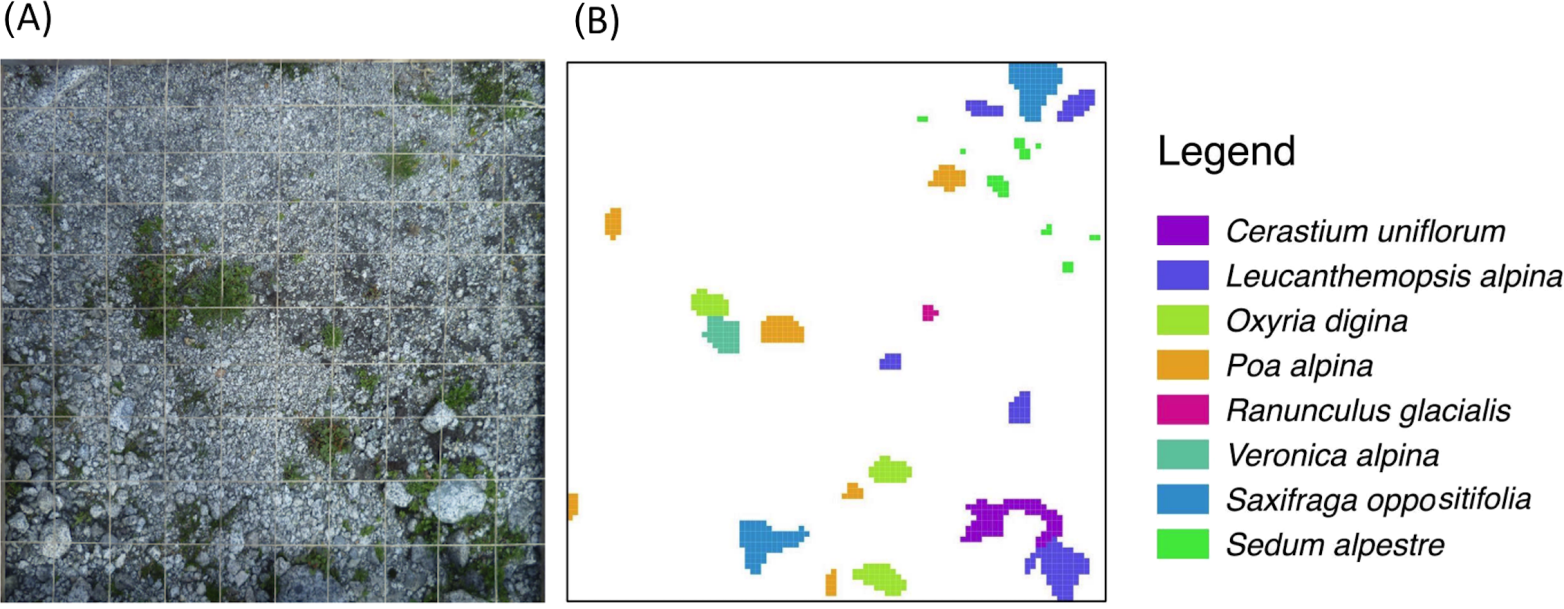
Schematic representation of the plant species survey. To each 1 m^2^ sampling plot (A) a virtual 1 cm-grid is superimposed to produce the final species-patch map (B).

We sampled 46 randomly distributed points in the study area. Using the closest individual method (Krebs, 1999), from each of these sampling points we selected the closest 1m × 1m sample plot, which was (i) safe from landslides and flat (<5°) and (ii) without boulders (*d* > 256 mm). This selection procedure was done to avoid marked differences in site conditions (Vetaas, 1997), even if it were not possible to completely control for a certain amount of variability in topsoil texture. Using historical maps and aerial photographs, frontlines of the glacier tongue, i.e., lines of equal terrain age (isochrones), were established for 1945, 1954, 1970, and 1996. Terrain ages (ta) of each sample plot were then classified as follows: (ta_1_) between 15 and 41 years (*n* = 11); (ta_2_) between 41 and 57 years (*n* = 17); and (ta_3_) between 57 and 66 years (*n* = 18) (Fig. 1C). Each plot was subdivided into 10,000 1 cm × 1 cm-grid cells. In August 2011, vascular plant species distribution intersecting the central axes of each 1 cm × 1 cm-cell were then mapped and digitised using ESRI ArcGIS 9.3, yielding for each species the number of 1 cm^2^ cells occupied per 1 m^2^ (Fig. 2). The same was done to classify the topsoil texture since, amongst the environmental variables, topsoil texture was considered to be a major physical influence on patch development. As a measure of topsoil texture we used the cover of cobbles, which, according to the Wentworth scale (Wentworth, 1922), have a diameter >64 mm, a size (>32 cm^2^) comparable to the observed mean size of plant patches (47 cm^2^). Then, we distinguished three classes of cobble cover: (ts_1_) <7% (*n* = 15); (ts_2_) between 7% and 14% (*n* = 15); and (ts_3_) >14% (*n* = 16).

### Data analysis

Landscape metrics were calculated according to the procedure of Teixido et al. (2007). For each 1 m × 1 m-plot, we calculated seven metrics (Table 1). Calculations were made using the Patch Analyst 5.0 extension for ArcGIS 9.3 (Rempel, Kaukinen & Carr, 2012), adopting a four-neighbour rule to identify the patches. A synthetic description of each patch metric is reported in Table 1. Two metrics, patch size and shape, can be computed both for every patch in the landscape and as an average for all patch metrics at the plot level (see also McGarigal & Marks, 1994). In the first case, the metrics may be related to each species, i.e., species-specific traits.

**Table 1:** Landscape metrics used to quantify plant patches patterns on a glacier foreland and plant species traits used to correlate them to the physiological characteristics of plant species.

Abbreviation	Unit	Variable name	Description
Landscape metrics			
MPS	cm^2^	Mean patch size	Mean size of all patches
PSCV	%	Patch size coefficient of variation	Variability in patch size relative to mean patch size. PSCV = 0 when all patches are the same size or when there is only 1 patch
TE	cm	Total edge	Total length of edge of all patch boundaries
NP	None	Number of patches	Total number of patches
MSI	None	Mean shape index	Mean shape index of all patches. MSI = 1 when a patch is maximally compact (i.e., a square) and increases without limit as patch shape becomes more irregular (Patton, 1975)
PR	None	Patch type richness	Number of different patch types (i.e., plant species)
SHDI	None	Shannon’s diversity index	−1 times the sum across all patch types, of the proportional abundance of each patch type multiplied by that proportion. SHDI = 0 when PR = 1 and increases without limit as PR increases and/or the proportional distribution of area among patch types becomes more equitable
Functional traits			
CH	mm	Canopy height	Aspects of competitive ability
LDMC	%	Leaf dry matter content	Resistance to physical hazards, long life-span, relative growth rate
LS	cat. (1–6)	Lateral spread	Aspects of competitive ability
LDW	mg	Leaf dry weight	Growth index
SLA	mm^2^ mg^−1^	Specific leaf area	Internal resistance to CO_2_ movement, nitrogen mass fraction, Rubisco specific activity, relative growth rate
LNC	%	Leaf nitrogen content	Assimilation capacity
LA	mm^2^	Leaf area	Leaf energy and water balance
LFW	mg	Leaf fresh weight	Growth index
LCC	%	Leaf carbon content	Photosynthetic rate

**Table 2:** Frequency, plant traits database (from different sources) and landscape metrics measured in the field on 46 1 m × 1 m plots. CA is the total cover in cm^2^. Standard deviation (±) are reported only for metrics which can be computed for every patch. Definitions, abbreviations and units of measurement of the landscape metrics are reported in Table 1. See Fig. 5 for abbreviations of species.

Species	Frequency (%) (*N* = 46)	Plant traits									Landscape metrics				
		CH	LDMC	LS	LDW	SLA	LNC	LA	LFW	LCC	MPS	CA	MSI	NP	TE
AdeLeu	2	358	17.1	3	116.5	21.4	3.2	2440.2	701.6	46.7	11	11	1.05	1	14
CarRes	11	24	26.0	2	3.7	16.2	3.6	58.2	14.4	45.4	9 ± 4	70	1.09 ± 0.07	8	100
CerUni	43	16	16.2	3	2.2	37.6	3.4	84.2	13.9	41.8	55 ± 151	2,318	1.23 ± 0.23	42	1,188
GeuRep	46	75	27.4	6	67.4	8.3	3.0	566.4	249.8	47.5	38 ± 41	2,717	1.19 ± 0.10	72	1,928
GnaSup	7	12	37.3	4	0.7	25.1	1.9	17.5	2.0	47.7	11 ± 7	128	1.16 ± 0.07	12	172
HieAlp	4	16	16.2	3	8.0	15.2	1.6	121.8	32.4	46.0	15 ± 15	30	1.21 ± 0.05	2	34
LeuAlp	70	33	16.4	4	2.4	18.2	2.6	42.4	15.2	44.7	40 ± 237	6,880	1.72 ± 0.20	173	4,018
LuzAlp	22	152	17.9	4	13.9	29.1	2.9	388.2	77.3	46.9	110 ± 222	2,086	1.25 ± 0.20	19	754
OxyDig	59	76	10.0	4	12.3	29.5	4.7	353.5	123.9	45.4	30 ± 38	6,206	1.26 ± 0.23	209	5,454
PoaAlp	96	36	34.3	3	29.6	11.1	1.3	328.6	86.6	45.8	24 ± 30	7,476	1.17 ± 0.12	310	6,456
RanGla	13	46	14.2	2	18.8	14.1	1.7	262.1	132.0	44.3	10 ± 7	177	1.1 ± 0.07	17	228
SalHer	15	14	33.2	6	7.6	15.2	2.0	165.0	22.9	47.4	307 ± 458	3,682	1.34 ± 0.35	12	1,042
SaxBry	72	5	32.4	4	0.2	15.3	1.8	2.8	0.7	44.0	95 ± 238	30,378	1.28 ± 0.27	320	13,324
SaxOpp	39	6	25.5	4	0.3	15.7	1.8	4.0	1.0	46.8	69 ± 114	5,702	1.25 ± 0.17	83	3,006
SedAlp	33	15	7.8	2	0.4	14.2	1.1	5.7	5.1	41.5	7 ± 6	644	1.13 ± 0.10	87	1,010
VerAlp	54	46	18.0	2	2.1	28.6	1.6	57.9	11.9	47.4	15 ± 14	2,013	1.17 ± 0.13	134	2,260

We did not sample plant traits in the field because this would have been too time-consuming and too destructive to the vegetation. When available, plant traits were taken from Cerabolini et al. (2010). Data for *Salix herbacea* L. and, partially, *Sedum alpestre* Vill. data were taken from Carbognani (2011) and Pierce et al. (2007). Leaf nitrogen and carbon contents of *S. alpestre* were calculated as a mean of the values of *Sedum acre* L. and *Sedum album* L. from Cerabolini et al. (2010). *Saxifraga stellaris* L., *Polygonum viviparum* L. and *Athyrium distentifolium* Tausch ex Opiz, due to their negligible frequencies and cover, were omitted from further analyses. A brief description of each selected trait is provided in Table 1 (see also Cornelissen et al., 2003; Gross, Suding & Lavorel, 2007; Wilson, Thompson & Hodgson, 1999). The plant traits database used here is reported in Table 2.

The 46 vegetation relevés were subjected to agglomerative cluster analysis using the Bray–Curtis coefficient of dissimilarity and Ward’s clustering method. Next, ANOVA was applied to verify for differences in landscape metrics between (i) the three classes of terrain age and (ii) topsoil texture distinguished, and (iii) the three floristic relevé clusters identified.

To relate plant traits to spatial configuration, taking into account species cover in the plots, we applied RLQ-analysis, a tool to assess how the environment filters certain species traits (Dolédec et al., 1996; Dray, Chessel & Thioulouse, 2003). The RLQ procedure performs a double inertia analysis of an environmental-variables-by-samples (R-table) and a species-by-traits (Q-table) matrix, with a link expressed by a species-cover-by-samples matrix (L-table). RLQ-analysis combines three unconstrained separate ordinations, correspondence analysis of L-table and centred normed principal component analyses of Q- and R- tables, to maximise the covariance between environmental factors and trait data by the use of co-inertia analysis (Bernhardt-Römermann et al., 2008). Here, we studied the joint structure of three data tables, namely (i) a plot-by-landscape metrics data table R-table, (ii) a plot-by-species table containing the abundances of the plant species present in our set of 46 plots table (L-table), and (iii) a species-by-trait data Q-table.

This RLQ analysis (basic-RLQ) was followed by a partial-RLQ, with the aim of checking the effects of the covariates terrain age and cobble cover, i.e., to verify the need to partition the variation related to these factors. This type of analysis is a special case of RLQ, where the covariable represents a partition of samples into groups. If the percentage of co-inertia explained by the most representative axis of partial-RLQ were to be much higher than in the basic-RLQ, this would mean that the influence of the covariate is relevant. The same approach was followed by Wesuls et al. (2012) to partition the response of plant traits to grazing-related environmental parameters from other environmental and temporal variations.

A permutation method was used to compare the hypothesis H_0_: *X* = 0 (trait and landscape are unrelated) against H_1_: *X* ≠ 0 (trait and landscape are related), where *X* is the fourth corner, a trait-by-landscape table, whose parameters cross the traits (Q-table) to the landscape variables (R-table), via the abundance table L-table (Legendre, Galzin & HarmelinVivien, 1997). The null hypothesis consists of three null joint hypotheses: both R and Q are linked to L (L ↔ Q, L ↔ R), only R is linked to L (L ↮ Q, L ↔ R), only Q is linked to L (L ↔ Q, L ↮ R). The overall null hypothesis is rejected when both null hypotheses are rejected (L ↮ Q and L ↮ R). Dray & Legendre (2008) proposed to set the alpha argument to $\alpha =\sqrt{0.05}$, but recently it has been shown that *α* should be 0.05 instead (Ter Braak, Cormont & Dray, 2012). Given the limited power of this test with few species (Ter Braak, Cormont & Dray, 2012), as in the present study, we show the results according to both the Dray & Legendre (2008) and the Ter Braak, Cormont & Dray (2012) alpha argument settings. A multivariate permutation test was applied to evaluate the global significance of the traits-spatial configuration relationships, implemented by the function ‘randtest’ of the package ade4 ( Dray & Dufour, 2007). Next, we tested the associations of spatial configuration and trait variables with the axes of the basic-RLQ. The strength of the association of landscape metrics and plant traits was measured with the D2 statistic (Dray et al., 2014). All tests were performed using the combined fourth-corner statistic (Dray et al., 2014) with 49,999 permutations.

All statistical analyses were performed using the open source R software (R Core Team , 2013). We used the library vegan (Oksanen et al., 2011) for the cluster analysis, the library stats (R Core Team , 2013) for ANOVA and the library ade4 ( Dray & Dufour, 2007) for the RLQ analysis.

## Results

### Species composition

We recorded a total of 19 plant species (including *S. stellaris*, *P. viviparum* and *A. distentifolium* which were omitted from further analyses). Each species frequency and mean patch-level metrics are reported in Table 2. This table shows that the most frequent species were *P. alpina*, *Saxifraga bryoides* L., *Leucanthemopsis alpina* (L.) Heywood, *O. digyna* and *Veronica alpina* L., present in more than half of the plots. Mean patch size, total cover and mean shape index of species did not necessarily reflect their frequency. Considering only the species where the number of sampled patches was higher than 30, the most compacted shapes belonged to *S. alpestre*, the most irregular to *L. alpina*, with the largest patches to *S. bryoides* and the smallest to *S. alpestre*. The dispersion of patch size values around the mean was much larger than for patch shape. The shape of the frequency distribution of patch sizes was usually positively skewed (Fig. S1), while patch shape values showed more frequently symmetrical distributions (Fig. S2).

**Figure 3:**
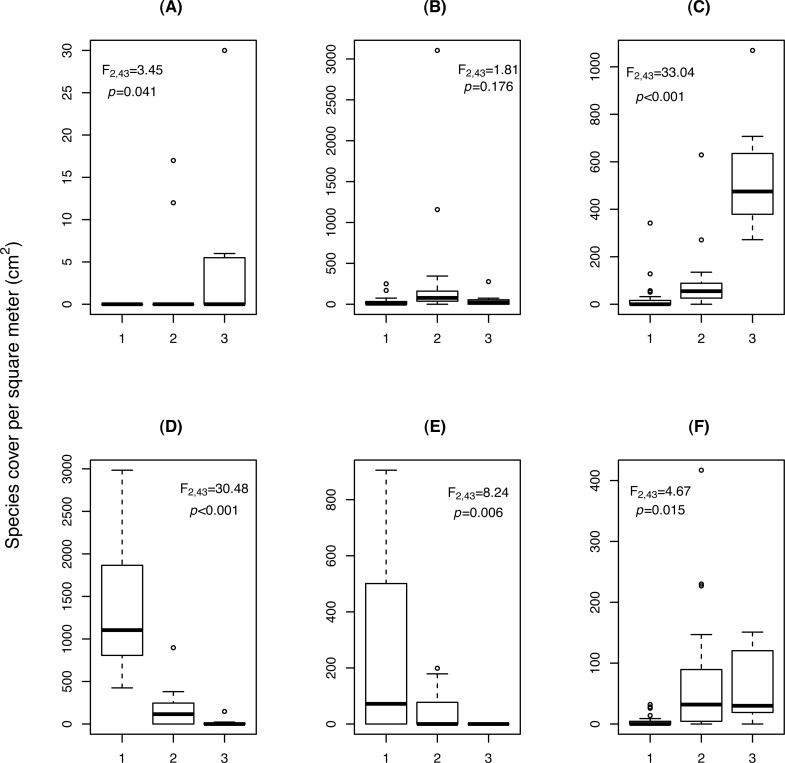
Change in cover (cm^2^/m^2^) of (A) *Cardamine resedifolia*, (B) *Leucanthemopsis alpina*, (C) *Oxyria digyna*, (D) *Saxifraga bryoides*, (E) *Saxifraga oppositifolia*, (F) *Veronica alpina,* among the three relevé clusters (1: *Saxifraga*-cluster, CL_1_; 2: *Leucanthemopsis*-cluster, CL_2_; 3: *Oxyria*-cluster, CL_3_). F and *p*-values were obtained by ANOVA.

Three clusters (CLs) resulted from the analysis of the 46 vegetation relevés, characterised by differences in the cover of six plant species (Fig. 3). In the *Saxifraga*-cluster (CL_1_), *S. oppositifolia* was much more abundant and *V. alpina* much less abundant than in the other two clusters; while in the *Leucanthemopsis*-cluster (CL_2_), *L. alpina* was more abundant. Finally, the *Oxyria*-cluster (CL_3_) was characterised by relatively high covers of *O. digyna* and *Cardamine resedifolia* L. (Fig. 3). ANOVA showed that the floristically defined relevé clusters differed significantly with regard to certain landscape metrics. The Shannon’s index of diversity and the patch type (=species) richness were significantly higher in the *Leucanthemopsis*-cluster (CL_2_) than in the *Saxifraga*-cluster (CL_3_) and the *Oxyria*-cluster (CL_1_) (Table 3). Also, the mean size of the vegetation patches differed significantly among the three relevé clusters, being greatest in the *Saxifraga*-cluster (CL_1_) and smallest in the *Leucanthemopsis*-cluster (CL_2_).

As expected, none of the landscape metrics differed amongst the three classes of terrain age, that is 15–41 y, 41–57 y and 57–66 y, and of cobble cover, that is <7% , between 7% and 14%, and >15% (results reported in Table S1). Correlations among landscape traits are reported in Table S2.

### Relationships between plant traits and landscape metrics

**Figure 4:**
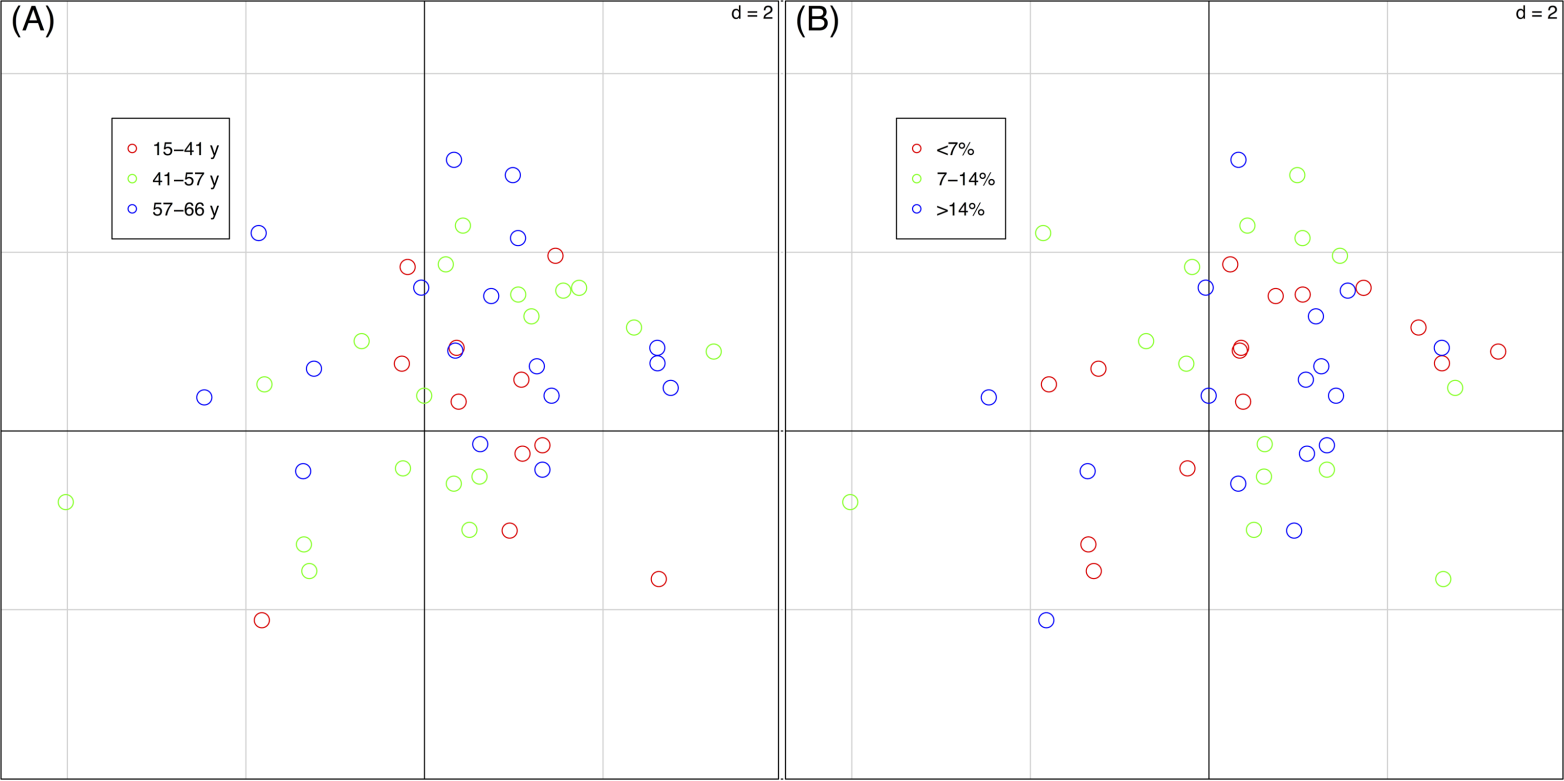
Sample scores (46 vegetation plots) of the first two axes of the basic-RLQ. The colours show the terrain age (A) and the cobble cover (B) classes of the sample plots. The eigenvalues are reported in Table 4. The values of d give the grid size.

The percentages of total co-inertia explained by the first two axes of the basic-RLQ was 96.1% while those of the two partial-RLQs were 95.3% and 95.7%, respectively adopting terrain age and cobble cover as covariates. The first axis of the basic-RLQ explained 89.8% co-inertia, while the percentage explained by the first axis of the two partial-RLQs was lower or almost identical (86.2% and 90.5%, same order above), meaning that the spatial configuration gradient along the first axis of both the partial-RLQs was not more pronounced than the basic-RLQ (Table 4). Moreover, the ordination diagrams of the basic-RLQ did not show any grouping of plots according either to the terrain age nor to the cobble cover factor (Fig. 4).

**Table 3:** Mean (±95% confidence intervals) of landscape metrics in relation to relevé clusters. Relevé clusters: (i) *Saxifraga*-cluster (CL_1_); (ii) *Leucanthemopsis*-cluster (CL_2_); and (iii) *Oxyria*-cluster (CL_3_). See Table 1 for abbreviations of landscape metrics. *P*-values were obtained by ANOVA. Statistically significant differences amongst groups are indicated in bold.

	MPS	PSCV	TE	NP	MSI	SHDI	PR
CL_**1**_	74.4 ± 10.1	190.3 ± 17.2	1,008 ± 63	32.0 ± 3.6	1.23 ± 0.01	0.82 ± 0.10	5.3 ± 0.4
CL_**2**_	36.9 ± 9.2	163.2 ± 22.1	807 ± 82	35.8 ± 3.0	1.19 ± 0.01	1.38 ± 0.07	6.5 ± 0.5
CL_**3**_	41.7 ± 5.2	118.6 ± 33.9	798 ± 158	26.1 ± 4.7	1.28 ± 0.03	1.11 ± 0.12	5.7 ± 0.7
F	5.108	2.059	2.41	1.328	9.946	11.39	1.915
*p*	**0.010**	0.14	0.102	0.275	**0.006**	**<0.001**	0.160

**Table 4:** Eigenvalues and percentage of variance (%) explained by the first five axes of the basic RLQ (sum of eigenvalues: 1.175) and the partial RLQs (sum of eigenvalues: 0.930 and 1.13, respectively using terrain age and cobble cover as covariates).

Axis	Basic RLQ		Partial RLQ terrain age		Partial RLQ cobble cover	
	Eigenvalue	%	Eigenvalue	%	Eigenvalue	%
1	1.056	89.8	0.802	86.2	1.023	90.5
2	0.075	6.3	0.085	9.1	0.059	5.2
3	0.029	2.4	0.027	2.9	0.027	2.3
4	0.010	0.8	0.012	1.3	0.011	1.0
5	0.005	0.4	0.002	0.2	0.009	0.8

The test for the model (H_1_: L ↔R) showed that the distribution of species with fixed traits was influenced by the spatial configuration (*p* = 0.004), while the test for the model (H_1_: L ↔Q) showed that species composition of plots with fixed spatial configuration was not influenced by the species traits (*p* = 0.590). This means that the traits-spatial configuration relationships were not globally significant.

The first basic-RLQ axis was significantly and negatively correlated with mean patch size and patch size coefficient of variation and positively to Shannon’s diversity. The second basic-RLQ axis was negatively correlated to mean shape index. Among the plant traits, canopy height, leaf fresh and dry weight and leaf area showed a positive significant correlation with the first basic-RLQ axis (Table 5).

**Figure 5:**
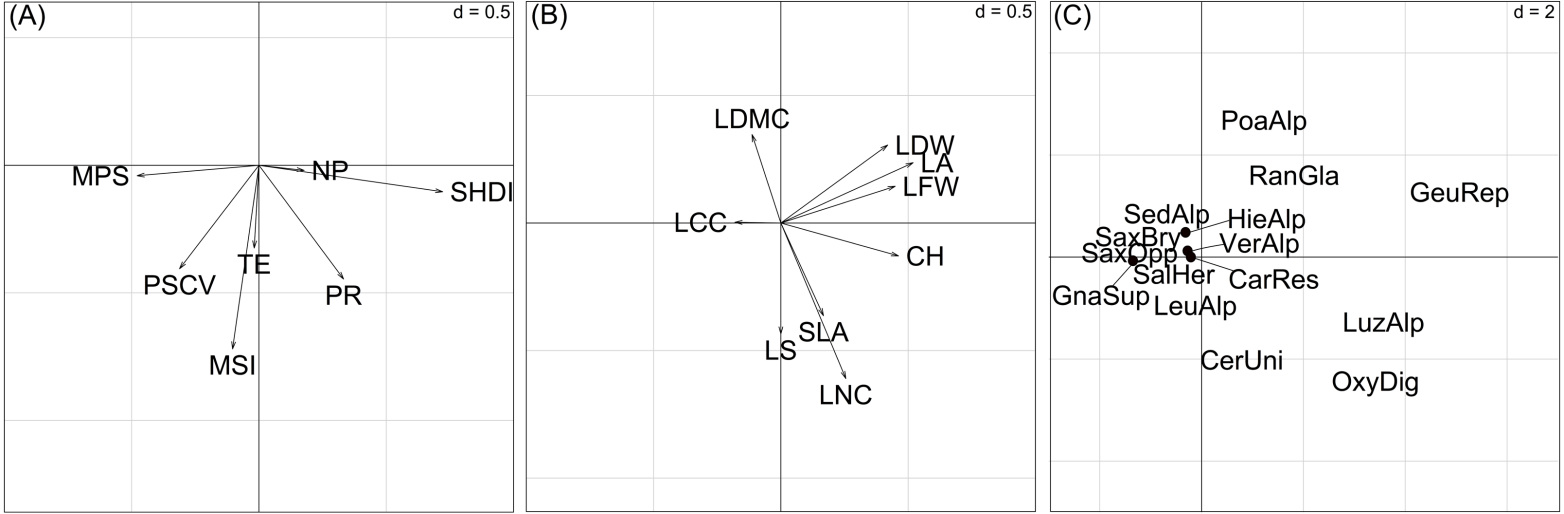
Ordination diagrams of the first two axes of the RLQ-analysis displaying the (A) landscape metrics scores, (B) plant trait scores, (C) species scores. Abbreviations for landscape metrics and plant traits are reported in Table 1. Abbreviations for species: CarRes, *Cardamine resedifolia*; CerUni, *Cerastium uniflorum*; GeuRep, *Geum reptans*; GnaSup, *Gnaphalium supinum*; HieAlp, *Hieracium alpinum*; LeuAlp, *Leucanthemopsis alpina*; LuzAlp, *Luzula alpino-pilosa*; OxyDig, *Oxyria digyna*; PoaAlp, *Poa alpina*; RanGla, *Ranunculus glacialis*; SalHer, *Salix herbacea*; SaxBry, *Saxifraga bryoides*; SaxOpp, *Saxifraga oppositifolia*; SedAlp, *Sedum alpestre*; VerAlp, *Veronica alpina*. *Adenostyles leucophylla* was not reported to avoid excessive gathering of points; its position is approximately (20, 5). The values of d give the grid size.

**Table 5:** Percentage contribution to total inertia of the basic RLQ and Pearson correlations of spatial configuration and plant functional traits with first two basic RLQ axes. Projected inertia by each axis is reported in parentheses. Associations significantly correlated with RLQ axes are shown in bold ($p\lt \sqrt{0.05}$) as proposed by Dray & Legendre (2008) or with an asterisk (*p* < 0.05) as proposed by Ter Braak, Cormont & Dray (2012). Abbreviations are reported in Table 1.

Variables	Contribution to total inertia (%)	Axis 1 (89.8%)	Axis 2 (6.3%)
*Spatial configuration*			
MPS	23.1	**−0.26**	−0.01
PSCV	26.2	**−0.17**	−0.08
NP	3.2	−0.09	0.00
TE	10.5	−0.01	−0.07
MSI	52.9	−0.06	**−0.15**
SHDI	53.1	**0.39**	−0.02
PR	31.0	0.18	−0.09
*Plant traits*			
CH	23.0	0.30 *	−0.03
LDMC	13.3	−0.07	0.08
LS	18.9	0.00	−0.10
LDW	27.0	**0.27**	0.07
SLA	16.0	−0.11	−0.08
LNC	43.7	0.16	−0.14
LA	32.6	0.34 *	0.05
LFW	22.2	0.29 *	0.03
LCC	3.2	−0.12	0.00

In summary, the first basic-RLQ axis represented a gradient of increasing diversity as a response to the presence of larger plant species patches (Fig. 5A). Moreover, as can be seen in Fig. 5B and Fig. 5C, the first basic-RLQ axis represented a gradient of increasing cover of taller species with larger and softer leaves, on the right hand side, like *Luzula alpino-pilosa* (Chaix) Breistr., *O. digyna*, *Geum reptans* L. and *Adenostyles leucophylla* (Willd.) Rchb., to tiny species with smaller, heavier and harder leaves on the left hand side, like saxifrages and *Gnaphalium supinum* L.. The second basic-RLQ axis, even if less pronounced than the first axis, represented a gradient of increasing conservation of acquired resources, that is from species with higher specific leaf area, leaf nitrogen content and tendency for lateral spread, like *Cerastium uniflorum* Clairv. and *O. digyna*, to species with larger dry matter content, like saxifrages (Fig. 5C). Interestingly, this axis also represents a gradient of increasing patch compactness.

**Figure 6:**
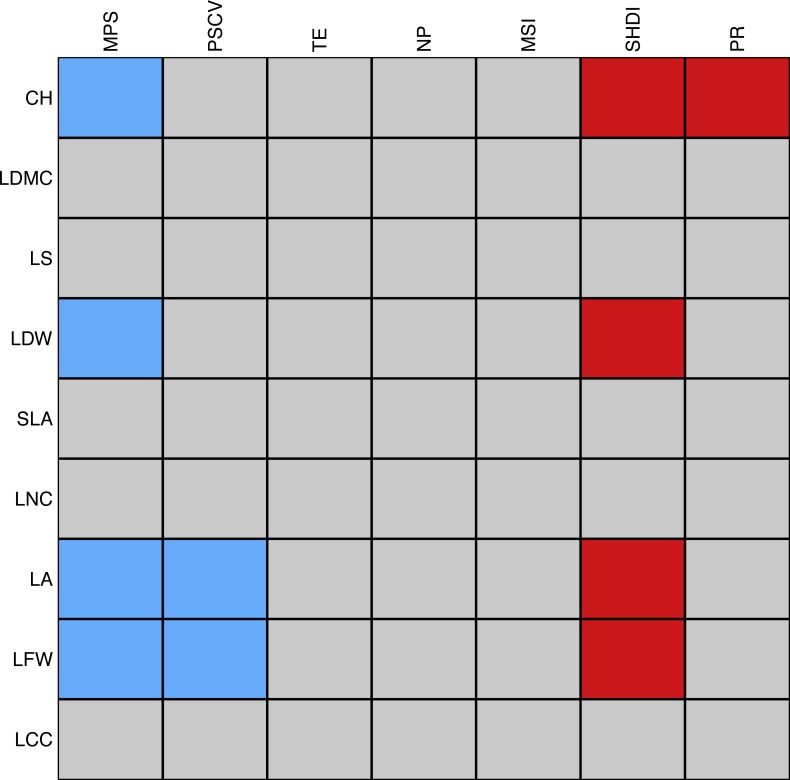
Schematic representation of the association between individual plant traits and landscape metrics. Red cells correspond to positive significant relationships while blue cells correspond to negative significant relationships. The strength of the association was measured with the D2 statistic and tested through a fourth-corner analysis (Dray et al., 2014). *P*-values were corrected by a sequential procedure (49,999 repetitions) which leads to significant associations if the maximum *p*-value was lower than $\alpha =\sqrt{0.05}$ as proposed by Dray & Legendre (2008). Abbreviations are reported in Table 1.

There were eleven significant associations between plant traits and landscape metrics. Mean patch size was negatively correlated with canopy height, leaf dry and fresh weight, and leaf area, the latter two of which were also negatively correlated with the coefficient of variation of patch size. Among the spatial metrics, the Shannon’s index of diversity was most frequently and positively associated with plant traits, namely with canopy height, leaf dry and fresh weight, and leaf area. Finally, patch richness was positively associated with canopy height (Fig. 6). We should stress that the majority of the tests were significant only when using a significance level of $\sqrt{0.05}$. The complete list of correlations between landscape and plant traits is reported in Table S3.

## Discussion

Prior work on glacier forelands has documented that whilst terrain age may appear to be one of the most obvious factors affecting plant colonisation processes (Caccianiga & Andreis, 2004), spatial patterns of plant species and their constituent assemblages are often heterogeneous and complex and not always directly related to the time factor (Burga et al., 2010; Matthews & Whittaker, 1987; Vetaas, 1994).

Here we applied a grid-sampling technique where the variables of interest are surveyed on a regular lattice grid at the appropriate scale. This approach, which has many possible applications, such as habitat suitability assessment (Sitzia et al., 2014a) and trail alignment (Sitzia et al., 2014b), has been possible since in the type of habitat studied here plant species form discrete patches in the form of small cushions, tussocks or rosettes separated by bare substrate.

### Species composition and landscape metrics

The terrain age and plant composition observed here correspond to early-successional stages of glacier foreland colonization; it does not include any mid- or late-successional species according to Caccianiga et al. (2006), apart from *S. bryoides*. However, this species, according to same authors, is present in the same proportion in early- and mid-successional sites. The composition also includes several ubiquitous species, like *P. alpina*, *L. alpino-pilosa* and *Hieracium alpinum* L. These species are typical wind-dispersed ruderals. The spectrum of plant traits encountered is limited and only some species, namely *A. leucophylla*, *P. alpina*, *Ranunculus glacialis* L., *G. reptans*, *L. alpino-pilosa*, and *O. digyna* exhibited relatively strong departures from the average characteristics of the main group of species.

We have shown that relevé clusters differ in landscape metrics. For instance, *S. bryoides* a species with small leaves but relatively high nitrogen content, showed the highest patch sizes amongst the frequent species and characterised a cluster with a correspondingly higher mean patch size. *V. alpina* and *L. alpina*, both medium-sized species, were present with many patches and, accordingly, characterised a cluster with a higher Shannon’s diversity. Finally, *O. digyna*, a relatively low compact species, with medium lateral spread and nitrogen content, was the most frequent in the cluster with the highest mean patch shape.

### Spatial configuration as a correlate of plant traits

The RLQ-analysis showed that plant species with efficient conservation of nutrients, which have a higher dry matter content in leaves (Pierce et al., 2013; Wilson, Thompson & Hodgson, 1999), also have lower values of the shape index, or, in other words, their patches are more compact. This pattern, maintained due to processes of intra- and interspecific competition for space and nutrients, ensures efficient acquisition-conservation trade-offs in plants characterized by slow growth, as in *P. alpina*, which, among the common species, is second in patch compactness only to *S. alpestre*. Second, we found that Shannon diversity increased with increasing cover of upright-growing plant species, characterised by larger and heavier leaves. A possible explanation for this lies in the way plants with these traits compete directly and/or indirectly, and how they modify one another’s biotic and abiotic environment, thereby generating a more equitable distribution of patch sizes, combined with a higher number of species.

The observed correlation of landscape metrics with species composition together with the correlations with specific life-form traits seem to indicate some level of life-form or species-based spatial self-organisation. Self-organization does not imply specific causalities between vegetation patterns and the environment, but is induced by internal variation independent of external drivers (Bolliger, Sprott & Mladenoff, 2003). Initial establishment of any particular species in this microhabitat depends on successful seed establishment. The first selection of species is mainly trait-driven because only species with a wind dispersal strategy are selected from the geographical species pool (Keddy, 1992). However, the later establishment is dependent on random abiotic factors such as wind-aided dispersal and by small-scale variation of the soil surface characteristics, such as texture, aiding germination. Stochastic factors thus potentially lead to a high degree of heterogeneity in seedling distribution due to the variability of seed rain, the soil seed bank, germination, mortality rates of the seedlings as well as other factors (Erschbamer, Kneringer & Schlag, 2001; Marcante, Winkler & Erschbamer, 2009). Large-scale successional stages usually contain a wide array of different microhabitats where species composition is mainly driven by habitat conditions and local disturbances (e.g., floods, rock falls, and avalanches) (Burga et al., 2010).

Here we tried to maintain, at a minimum level, these potential environmental filters by selecting a restricted range of terrain ages, slope, and topsoil texture. This has probably favoured us in finding the observed relationships between landscape patch metrics and functional traits.

### Landscape metrics as functional traits

Landscape-scale variation, usually surveyed at larger resolutions than those used here, is often seen as a filter of plant traits rather than a plant trait itself. This can be partially explained by the need to study how environmental change and disturbance, especially anthropogenic, may affect the landscape mosaic and in turn filter specific traits (Gámez-Virués et al., 2015). Another possible explanation is the difficulty to select the appropriate grain and extent to correctly detect each species’ patch boundaries. According to O’Neill et al. (in Turner, Gardner & O’Neill, 2001) the grain size of a map should be two to five times smaller than the spatial features being analysed, and map extent should be two to five times larger than the largest patches. The mean, upper quartile and maximum patch size was 47 cm^2^, 35 cm^2^, and 3,104 cm^2^, respectively. This means that the extent (1 m^2^) we chose was appropriate and that the grain (1 cm^2^) could be apparently increased. However, some species presented very low mean patch sizes, e.g., *S. alpestre* (only 7 cm^2^). This means that to perform a multi-species patch-level calculation of landscape metrics on a glacier foreland with terrain ages <70 y, the grain should be maintained at least lower than 3.5–1.4 cm^2^, in practice, units of 3.0–1.0 cm^2^. Finally, we have to stress again that the identification of each patch could be difficult in late successional stages, where their edges could not be easily distinguished.

Databases of plant functional traits such as LEDA (Knevel et al., 2003), TRY (Kattge et al., 2011) or BIOPOP (Poschlod et al., 2003) are very useful since they provide possible determinants of the response of primary producers to environmental factors, and how they can affect other trophic levels, ecosystem processes, services and diversity (Kattge et al., 2011). Existing databases of functional traits do not include data about patch-level landscape metrics of plant species. We suggest that these databases should incorporate such data.

One possible criticism of this proposal might be that some landscape traits might already be incorporated in existing plant species attributes. However, this should be treated with caution. For example, while patch size might be associated with the Hodgson et al.’s (1999) lateral spread measure, included as a class parameter into the attribution of Grime’s (1977) CSR strategy, here we observed that a reduction in patch size is more clearly associated with canopy height and leaf traits. Also the capability to perform clonal growth, resulting in large and compact patches, is an attribute largely included into existing databases and is well known for many alpine grasses and sedges (Groenendael et al., 1996). On the other hand, patch size and shape could be linked to ontogenetic features of the individual (i.e., individual age) for species with indefinite growth such as many woody species, like many cushion species. Individual age, in turn, could be related to stochastic station parameters such as terrain age, which may be the case in a glacier foreland, and should not be considered as a plant trait. Compactness of the patch itself could be the result of many plant attributes and thus requires care if treated as a plant trait. Moreover, non-clonal single individuals are rather compact and we have shown that the most compact patches are also the smallest. This could be due to the situation where the species occurs in single individuals. In general, we have shown that the distribution of species-specific landscape trait values, in particular patch size, might be rather skewed, meaning the mean might not be the best summarising statistical indicator and that care should be taken in the selection of the individuals where measuring the traits (Cornelissen et al., 2003). All these aspects call for further research to collect these data and to link them with physiological attributes directly measured in the field.

## Conclusions

In summary, we have presented a new high-resolution approach for the detection of landscape-level correlation with plant species traits. Analyses of how species patches are spatially arranged in herbaceous glacier foreland communities indicate that diversity in patch types and size both increases whilst patch size decreases with increasing canopy height, leaf size and weight. Moreover, more compact patch shapes indicate an increased capacity of conservation of nutrients in leaves. We stress the need to perform further analyses on the distribution of species’ patch sizes, which here we nested inside square plots, but, treating the plots as random factors could provide further insights into the dynamics of space use and niche overlap on glacier forelands. Moreover, some of the results were not statistically significant because of the limited range of terrain age analysed and the few species surveyed. A wider span of terrain ages and, in turn, of plant traits, which would also consider the effect of time since deglaciation and environmental variability, would provide further information about the amount of the self-induced, trait-driven variability in spatial configuration and that related to allogenic, environmental filters, thus increasing statistical robustness.

Any measurable attribute at the individual level that directly or indirectly influence overall fitness or performance might be regarded a functional trait (Violle et al., 2007). According to this, we conclude that patch-level landscape metrics of plants should be treated as functional traits. Further research is needed in a range of ecosystems but we expect this approach to open up an entirely new class of plant traits, i.e., landscape plant traits, which should be collected at patch level and might bring additional functional meaning as predictors of life strategies.

##  Supplemental Information

10.7717/peerj.3552/supp-1 Figure S1 Frequency distribution of patch size of the most common plant species Frequency distribution of patch size of the plant species represented by at least thirty patches.

10.7717/peerj.3552/supp-2 Figure S2 Frequency distribution of patch shape of the most common plant species Frequency distribution of patch size of the plant species represented by at least thirty patches.

10.7717/peerj.3552/supp-3 Table S1 Mean (±95% confidence intervals) of landscape metrics in relation to terrain ages (A) and cobble cover (B) Terrain ages (ta) of each sample plot were classified as follows: (ta _1_) between 15 and 41 years (*n* = 11); (ta _2_) between 41 and 57 years (*n* = 17); and (ta _3_) between 57 and 66 years (*n* = 18). We distinguished three classes of cobble cover: (ts _1_) < 7% (*n* = 15); (ts _2_) between 7% and 14% (*n* = 15); and (ts _3_) > 14% (*n* = 16). See Table 1 for abbreviations of landscape metrics. *P*-values were obtained by ANOVA.

10.7717/peerj.3552/supp-4 Table S2 Pearson correlation coefficients for all possible pairs of landscape metrics Significant values (*P* < 0.05) are indicated in bold. See Table 1 for abbreviations of landscape metrics.

10.7717/peerj.3552/supp-5 Table S3 Results of the fourth-corner tests. Pearson correlation coefficients (*r*) for all possible pairs of landscape and plant traits are reported The significance (*p*) is tested by a permutation procedure. Code for traits and variables are explained in Table 1.

10.7717/peerj.3552/supp-6 Supplemental Information 1 R-table: plot-by-landscape metrics data table

10.7717/peerj.3552/supp-7 Supplemental Information 2 Q-table: species-by-trait data table

10.7717/peerj.3552/supp-8 Supplemental Information 3 L-table: a plot-by-species table containing the abundances of the plant species present in our set of 46 plots

10.7717/peerj.3552/supp-9 Supplemental Information 4 Database of the patches x species attribute

10.7717/peerj.3552/supp-10 Supplemental Information Terrain age, cobble cover and coordinates of each plot

## Additional Information and Declarations

### Data Availability

The following information was supplied regarding data availability: The raw data has been supplied as a Supplementary File.
