# Supplementary figures and images for: Landscape metrics as functional traits in plants: perspectives from a glacier foreland
Source: PeerJ. 2017 Jul 31;5:e3552. doi: 10.7717/peerj.3552 (PMC5541930; doi:10.7717/peerj.3552)

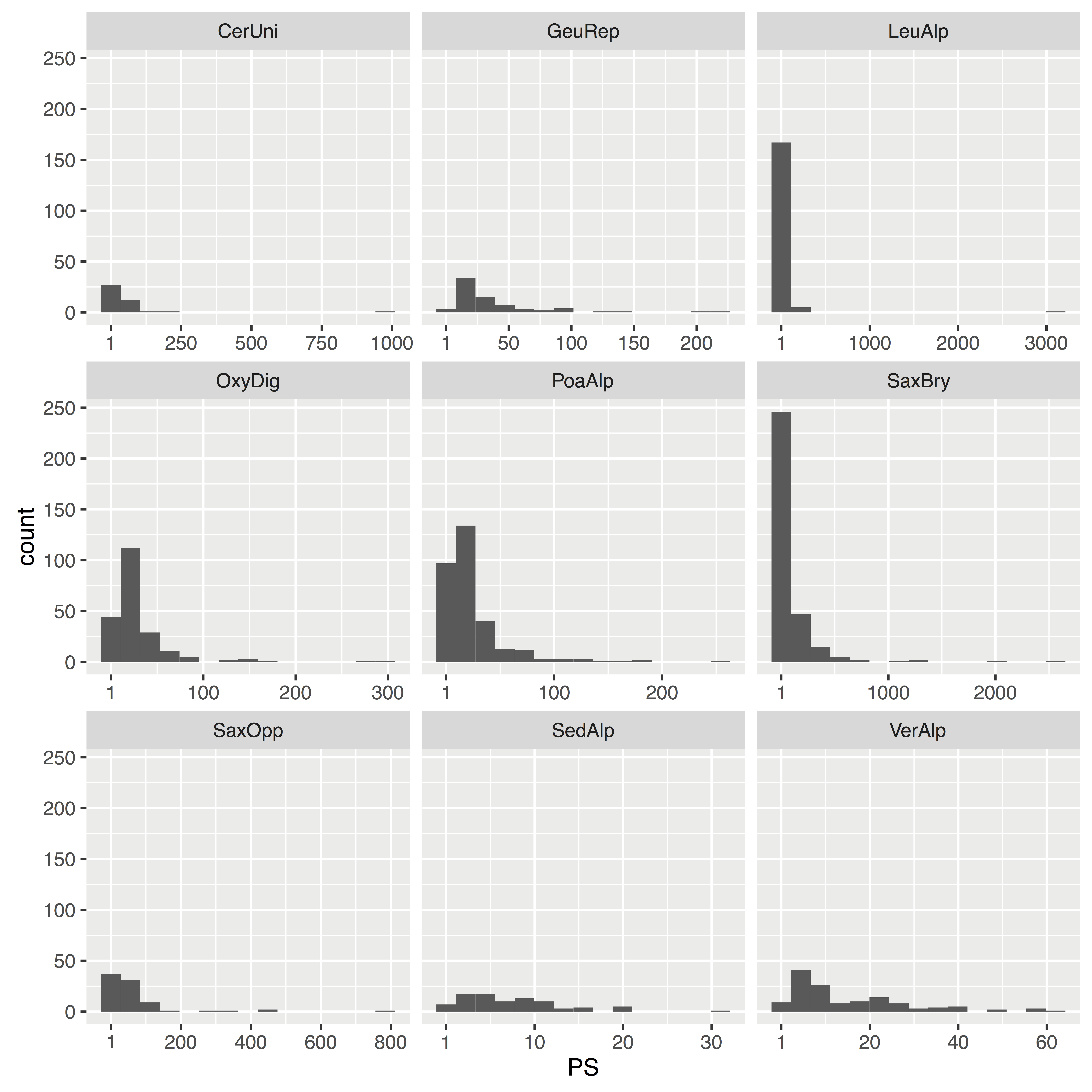

Supplement: Figure S1 — Frequency distribution of patch size of the plant species represented by at least thirty patches. [file peerj-05-3552-s001.png]
