# Supplementary material for: Landscape metrics as functional traits in plants: perspectives from a glacier foreland
Source: PeerJ. 2017 Jul 31;5:e3552. doi: 10.7717/peerj.3552 (PMC5541930; doi:10.7717/peerj.3552)
Supplement: Table S1 — Terrain ages (ta) of each sample plot were classified as follows: (ta 1) between 15 and 41 years (n = 11); (ta 2) between 41 and 57 years (n = 17); and (ta 3) between 57 and 66 years (n = 18). We distinguished three classes of cobble cover: (ts 1) < 7% (n = 15); (ts 2) between 7% and 14% (n = 15); and (ts 3) > 14% (n = 16). See Table 1 for abbreviations of landscape metrics. P-values were obtained by ANOVA. [file peerj-05-3552-s003.docx]

**Table S1**

Mean (± 95% confidence intervals) of landscape metrics in relation to terrain ages (a) and cobble cover (b). Terrain ages (ta) of each sample plot were classified as follows: (ta_1_) between 15 and 41 years (n=11); (ta_2_) between 41 and 57 years (n=17); and (ta_3_) between 57 and 66 years (n=18). We distinguished three classes of cobble cover: (ts_1_) < 7% (n=15); (ts_2_) between 7% and 14% (n=15); and (ts_3_) > 14% (n=16). See Table 1 for abbreviations of landscape metrics. *P*-values were obtained by ANOVA.

**a)**

|  | **MPS** | **PSCV** | **TE** | **NP** | **MSI** | **SHDI** | **PR** |
| --- | --- | --- | --- | --- | --- | --- | --- |
| **ta_1_** | 61.1 ± 11.2 | 177.5 ± 32.0 | 1035 ± 121 | 32.3 ± 3.3 | 1.22 ± 0.02 | 1.19 ± 0.10 | 6.8 ± 0.7 |
| **ta_2_** | 41.4 ± 9.2 | 171.1 ± 20.8 | 792 ± 79 | 34.7 ± 4.7 | 1.20 ± 0.02 | 1.10 ± 0.12 | 5.3 ± 0.5 |
| **ta_3_** | 61.4 ± 12.4 | 159.8 ± 40.6 | 902 ± 72 | 31.1 ± 2.8 | 1.24 ± 0.02 | 1.03 ± 0.12 | 5.8 ± 0.4 |
| **F** | 1.24 | 0.16 | 2.00 | 0.30 | 1.44 | 0.43 | 2.24 |
| ***p*** | 0.30 | 0.85 | 0.15 | 0.74 | 0.25 | 0.65 | 0.12 |

**b)**

|  | **MPS** | **PSCV** | **TE** | **NP** | **MSI** | **SHDI** | **PR** |
| --- | --- | --- | --- | --- | --- | --- | --- |
| **ts_1_** | 56.0 ± 10.4 | 165.7 ± 20.4 | 920 ± 86 | 33.9 ± 4.4 | 1.21 ± 0.02 | 1.10 ± 0.13 | 5.9 ± 0.5 |
| **ts_2_** | 55.3 ± 13.1 | 181.6 ± 23.7 | 848 ± 96 | 32.2 ± 4.4 | 1.20 ± 0.01 | 1.04 ± 0.13 | 6.1 ± 0.7 |
| **ts_3_** | 50.8 ± 11.1 | 158.0 ± 24.9 | 910 ± 84 | 32.1 ± 2.8 | 1.25 ± 0.02 | 1.14 ± 0.10 | 5.5 ± 0.3 |
| **F** | 0.07 | 0.31 | 0.22 | 0.08 | 2.08 | 0.22 | 0.43 |
| ***p*** | 0.93 | 0.73 | 0.80 | 0.92 | 0.14 | 0.81 | 0.65 |
