# Supplementary material for: Landscape metrics as functional traits in plants: perspectives from a glacier foreland
Source: PeerJ. 2017 Jul 31;5:e3552. doi: 10.7717/peerj.3552 (PMC5541930; doi:10.7717/peerj.3552)
Supplement: Table S2 — Significant values (P < 0.05) are indicated in bold. See Table 1 for abbreviations of landscape metrics. [file peerj-05-3552-s004.docx]

**Table S2**

Pearson correlation coefficients for all possible pairs of landscape metrics. Significant values (P<0.05) are indicated in bold. See Table 1 for abbreviations of landscape metrics.

|  | MPS | PSCV | TE | NP | MSI | SHDI | PR |
| --- | --- | --- | --- | --- | --- | --- | --- |
| MPS |  | **0.64** | **0.35** | **-0.41** | **0.51** | **-0.52** | -0.05 |
| PSCV | **0.64** |  | **0.44** | 0.05 | 0.12 | **-0.3** | 0.2 |
| TE | **0.35** | **0.44** |  | **0.55** | **0.32** | -0.01 | 0.42 |
| NP | **-0.41** | 0.05 | **0.55** |  | **-0.35** | **0.37** | **0.41** |
| MSI | **0.51** | 0.12 | **0.32** | **-0.35** |  | **-0.46** | -0.18 |
| SHDI | **-0.52** | **-0.3** | -0.01 | **0.37** | **-0.46** |  | **0.61** |
| PR | -0.05 | 0.2 | **0.42** | **0.41** | -0.18 | **0.61** |  |
