# Supplementary material for: Landscape metrics as functional traits in plants: perspectives from a glacier foreland
Source: PeerJ. 2017 Jul 31;5:e3552. doi: 10.7717/peerj.3552 (PMC5541930; doi:10.7717/peerj.3552)
Supplement: Table S3 — The significance (p) is tested by a permutation procedure. Code for traits and variables are explained in Table 1. [file peerj-05-3552-s005.docx]

**Table S2**

Results of the fourth-corner tests. Pearson correlation coefficients (r) for all possible pairs of landscape and plant traits are reported. The significance (*p*) is tested by a permutation procedure. Code for traits and variables are explained in Table 1.

| Pairs | r | *p* |
| --- | --- | --- |
| MPS / CH | -0.202 | 0.174 |
| PSCV / CH | -0.104 | 0.514 |
| TE / CH | 0.001 | 0.985 |
| NP / CH | 0.069 | 0.576 |
| MSI / CH | -0.056 | 0.743 |
| SHDI / CH | 0.365 | 0.129 |
| PR / CH | 0.194 | 0.180 |
| MPS / LDMC | 0.066 | 0.778 |
| PSCV / LDMC | 0.060 | 0.679 |
| TE / LDMC | -0.121 | 0.304 |
| NP / LDMC | -0.051 | 0.718 |
| MSI / LDMC | -0.084 | 0.460 |
| SHDI / LDMC | -0.080 | 0.912 |
| PR / LDMC | -0.029 | 0.882 |
| MPS / LS | 0.039 | 0.852 |
| PSCV / LS | 0.041 | 0.801 |
| TE / LS | 0.041 | 0.711 |
| NP / LS | 0.021 | 0.882 |
| MSI / LS | 0.067 | 0.540 |
| SHDI / LS | 0.010 | 0.973 |
| PR / LS | 0.084 | 0.635 |
| MPS / LDW | -0.203 | 0.192 |
| PSCV / LDW | -0.167 | 0.229 |
| TE / LDW | -0.016 | 0.927 |
| NP / LDW | 0.097 | 0.401 |
| MSI / LDW | -0.1190 | 0.386 |
| SHDI / LDW | 0.297 | 0.170 |
| PR / LDW | 0.113 | 0.441 |
| MPS / SLA | -0.101 | 0.658 |
| PSCV / SLA | 0.053 | 0.722 |
| TE / SLA | -0.031 | 0.810 |
| NP / SLA | 0.022 | 0.925 |
| MSI / SLA | 0.032 | 0.779 |
| SHDI / SLA | 0.1373 | 0.790 |
| PR / SLA | 0.121 | 0.557 |
| MPS / LNC | -0.126 | 0.556 |
| PSCV / LNC | -0.030 | 0.819 |
| TE / LNC | 0.031 | 0.865 |
| NP / LNC | 0.036 | 0.808 |
| MSI / LNC | 0.115 | 0.267 |
| SHDI / LNC | 0.203 | 0.638 |
| PR / LNC | 0.155 | 0.382 |
| MPS / LA | -0.263 | 0.090 |
| PSCV / LA | -0.199 | 0.085 |
| TE / LA | -0.055 | 0.614 |
| NP / LA | 0.085 | 0.471 |
| MSI / LA | -0.088 | 0.536 |
| SHDI / LA | 0.376 | 0.113 |
| PR / LA | 0.150 | 0.279 |
| MPS / LFW | -0.229 | 0.131 |
| PSCV / LFW | -0.196 | 0.102 |
| TE / LFW | -0.020 | 0.913 |
| NP / LFW | 0.078 | 0.499 |
| MSI / LFW | -0.048 | 0.816 |
| SHDI / LFW | 0.318 | 0.152 |
| PR / LFW | 0.126 | 0.375 |
| MPS / LCC | 0.093 | 0.711 |
| PSCV / LCC | 0.029 | 0.895 |
| TE / LCC | -0.046 | 0.701 |
| NP / LCC | -0.064 | 0.675 |
| MSI / LCC | 0.042 | 0.762 |
| SHDI / LCC | -0.145 | 0.757 |
| PR / LCC | -0.035 | 0.905 |
